# Challenging Diagnosis and Management: A Complex Case Following an Urgent Caeserean Section

**DOI:** 10.7759/cureus.49227

**Published:** 2023-11-22

**Authors:** Gabriela Tenreiro, Carolina Santos, Elvira Machado, Sandra Carneiro

**Affiliations:** 1 Anesthesiology, Unidade Local de Saúde de Matosinhos, Matosinhos, PRT

**Keywords:** aortic diseases, cardiac complications, procedural complications, obstetric anesthesiology, caesarean section

## Abstract

Hemodynamic changes during pregnancy are physiological adaptations to fulfill new demands. Although these adaptations are often well-tolerated, they may unmask or exacerbate underlying cardiovascular conditions, posing unique challenges for medical professionals. We present a case that outlines the evaluation and treatment of a 38-year-old woman who experienced health complications shortly after undergoing a Caesarean section.

A 38-year-old woman who had a previous caeserean section presented for an urgent caeserean section at 39 weeks of gestational age due to non-reassuring cardiotocography. Shortly after spinal anesthesia and misoprostol administration, she developed cyanosis, confusion, mottling skin, tachycardia, tachypnea, and hypotension. The patient's clinical presentation led to a broad differential diagnosis, including cardiovascular complications, infection, medication reactions, and hemorrhage, being the ultimate diagnosis of a case of severe aortic stenosis. Successful management involved a multidisciplinary approach and coordinated effort, particularly involving the anesthesiology team, which was pivotal in timely diagnosis and intervention.

The ultimate diagnosis of severe aortic stenosis emphasized the significance of accurate and coordinated healthcare. Successful management involves collaboration between obstetric, intensive care, and cardiac care teams, highlighting the potential for improved outcomes when healthcare professionals work together in complex clinical scenarios.

## Introduction

Pregnancy-related hemodynamic changes result from a complex interaction of physiological adaptations to fulfill the heightened demands of both mother and fetus. These changes include adjustments in cardiac output, vascular resistance, and volume status [1]. The expansion of blood volume and the rise in cardiac output are essential to provide oxygen and nutrients to the developing fetus [2]. Although these adaptations are often well-tolerated, they can unmask or exacerbate underlying cardiovascular conditions, posing unique challenges for medical professionals.

This case underscores the need for heightened awareness of the hemodynamic transformations during pregnancy and the clinical implications of cardiovascular conditions in expectant mothers. It delves deeper into the complex differential diagnosis that arises when a patient deteriorates following an urgent Caesarean delivery, highlighting the critical role that the anesthesiology team plays in both the diagnosis and treatment process.

We provide a case that details the process of diagnosis and treatment of a 38-year-old parturient who became unwell immediately after an urgent Caesarean section.

## Case presentation

A 38-year-old G2 P1 woman was proposed for an urgent caeserean section at 39 weeks of gestational age due to a non-reassuring cardiotocography. The patient had a previous Caesarean section four years prior due to breech presentation and had a history of migraines and a prolactinoma diagnosed at the age of 27. Her endocrinologist followed her throughout the pregnancy with stable prolactin values, and no medication was needed. The current pregnancy had been uncomplicated.

On a Sunday evening, the anesthesiology team was contacted because of signs of fetal distress. She was proposed for an urgent Caesarean section, although there were no signs of an immediate life threat. During the short preoperative physical examination, the 164-cm-tall, 83-kg pregnant patient had minor peripheral edema, a Mallampati class II airway, and a 4 cm thyromental space. Her vital signs were as follows: heart rate 102 bpm, respiratory rate 21 breaths/min, blood pressure 123/71 mmHg, oxygen saturation 98%, and a body temperature of 37°C. Preoperative laboratory examinations showed no obvious abnormalities. After a brief clinical history, it was confirmed that she had no allergies, her last meal was 8 hours prior, and no medications were administered before the procedure.

A combined spinal-epidural technique was proposed, and the parturient understood and accepted it. Informed consent was obtained. The operating room continuously monitored the electrocardiogram (ECG), pulse rate, oxygen saturation, and non-invasive blood pressure. She underwent spinal anesthesia in a sitting position at L4-L5 with 10 mg of 0.5% heavy bupivacaine, and then the epidural catheter was placed. The sensory block was at the T4 level, and she remained conscious and comfortable throughout the procedure. Approximately 10 minutes after the spinal administration, her blood pressure dropped to 97/54 mm Hg, and her heart rate decreased to 83 bpm. She also became intensely nauseated, so 5 mg of ephedrine was administered. Non-invasive blood pressure was subsequently monitored in intervals of 3 minutes. No additional administration of vasopressors was necessary as the patient remained with mean arterial pressures (MAP) above 65 mmHg.

A 3980 g male baby was born 16 minutes later with an Apgar score of 9-10. There was an estimated blood loss of 350 mL. Oxytocin, 10U, was administered intravenously, diluted in 1000 mL of 0.9 % saline solution as the uterotonic agent. After this, she experienced another episode of hypotension with associated symptoms, including lightheadedness and nausea, necessitating the administration of two more boluses of 5mg each of ephedrine for a MAP goal of a minimum of 65 mmHg.

The procedure finished without further complications, but due to poor uterine contraction, it was decided to rectally insert four misoprostol tablets at the end of the surgery. She was transferred to the anesthetic care unit, hemodynamically stable and without complaints.

About 5 minutes later, the patient became cyanotic, experienced extreme shivering, confusion, mottling skin, tachycardia of 129 bpm, tachypnea of 28rpm, and had a temperature of 38.7ºC. Due to shivering and poor peripheral perfusion, oxygen saturation and blood pressure could not be obtained. An arterial gasometry with FiO2 40% demonstrated the following values: pH 7.41, PaO2 71 mmHg, PaCO2 30 mmHg, HCO3- 28.1 mmol/L, SpO2 93%, lactates 4.1, and no ionic disturbances. The four tablets of misoprostol were immediately removed. Paracetamol and meperidine were administered, and active heating was initiated. The patient started to improve slowly. Systolic blood pressures remained slightly low with a MAP around 50 mmHg, so an infusion of phenylephrine was started, and fluid therapy was administered in a total of 2 liters of crystalloid fluid.

**Figure 1:**
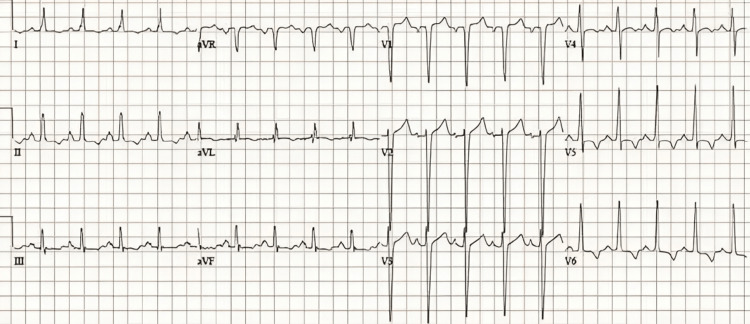
ECG after urgent procedure showing sinus tachycardia and T wave inversion

At this point, the main differential diagnoses included pulmonary embolism, amniotic fluid embolism, prostaglandin-induced reactions, sepsis, hemorrhage or peripartum cardiomyopathy. An ECG showed sinus tachycardia with T wave inversion in V5-V6 (Figure 1).

The results of the hematological and biochemical analyses were normal, except for a small increase in troponin I with a normal BNP. A pulmonary contrast tomography angiography excluded pulmonary embolism and revealed no significant alterations except a thin layer of pleural effusion.

**Figure 2:**
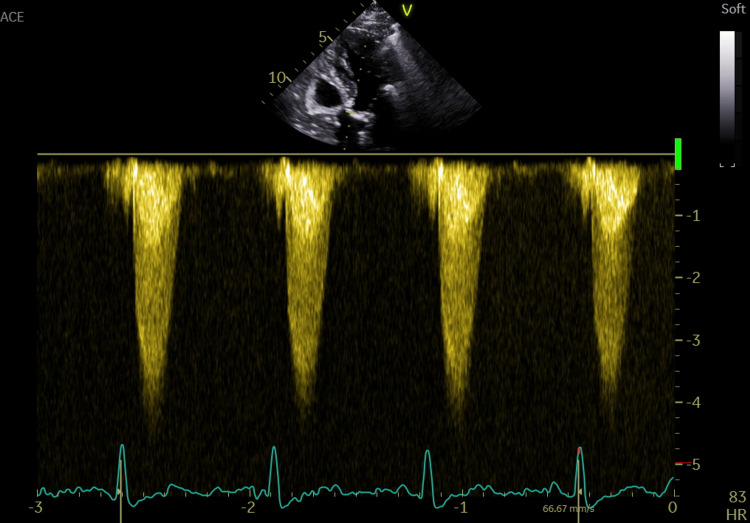
Apical 5-chamber view CW Doppler tracings showing a Peak velocity ≥ 4 m/s

After discussing the clinical case with the intensive care team, it was decided to contact the cardiology team that was on call. After reviewing the case, the cardiologist agreed that an urgent transthoracic echocardiogram (TTE) was necessary. The exam revealed a bicuspid aortic valve with severe stenosis, an effective orifice area (EOA) of 0.8 cm2, and a mean gradient of 58 mmHg (Figure 2, Video 1). Moderate functional mitral regurgitation and significant pulmonary hypertension (TAPSE 54 mmHg) were present. Moderate aortic insufficiency and mild impairment of ejection fraction (51%) were also noted. Cardiac cavities were within normal size, and there were no apparent changes in segmental contractility.

**Video 1:** Parasternal long axis showing calcificated valve with limited mobility of cusps

The patient was transferred to the intensive care unit, where she remained stable without the need for vasopressors or other specialized care. Subsequent discussions with the patient revealed that she had been experiencing precordial discomfort after moderate exertion with relief at rest, orthopnoea, and dyspnea on slight exertion in recent months, which she had attributed to her pregnancy. On physical examination, a grade IV systolic heart murmur was evident; however, there were no records of any changes to the cardiac auscultation in her previous medical file. The previous caeserean section went uneventful.

The Cardio-Thoracic Surgery department at the referral hospital was contacted, and on the 4th post-operative day, the patient was discharged home with an appointment scheduled for valve replacement surgery. About a month later, the patient underwent aortic valve replacement at the tertiary referral hospital without any major complications.

## Discussion

The differential diagnosis for a patient who develops signs of hypoperfusion following an urgent caeserean section is broad and must be approached with careful consideration [3]. The symptoms displayed by the patient, including cyanosis, confusion, mottling skin, tachycardia, tachypnea, and low blood pressure, can lead to an array of potential diagnoses, ranging from cardiovascular complications to infection, hemorrhagic complications and adverse reactions to medications.

The first alarm symptom we noticed was extreme shivering. Shivers can be attributed to a variety of causes, and it is frequent after a surgical procedure due to post-operative hypothermia, stress, anxiety, and pain. However, the extreme degree of shivering accompanied by the other shown symptoms is not so typical. Misoprostol is a synthetic prostaglandin E1 analog that causes uterine contractions, and one of its main uses is a uterotonic for the prevention of postpartum hemorrhage. One of its most common side effects is shivering and fever [4], so it was one of the first differential diagnoses, leading to the quick suspension of the drug in this case.

This case report highlights the central role of the anesthesiology team in the diagnostic process and the management of complex clinical presentations. As the patient's clinical condition deteriorated, promptly recognizing the situation's urgency and their coordinated efforts were crucial in ensuring a timely diagnosis and consequent intervention. In this case, the anesthesiology team evaluated the immediate symptoms and initiated appropriate measures.

A valuable takeaway from this case is the utility of transthoracic echocardiography (TTE) in postpartum patients who are unstable. Pulmonary embolism, amniotic fluid embolism, decompensated heart failure, and sepsis were among the differential diagnoses. TTE is a non-invasive technique that can swiftly differentiate between these diagnoses. The ultimate diagnosis of severe aortic stenosis, confirmed through the TTE, offered a comprehensive explanation for the patient's clinical presentation.

Research has indicated that heart failure in pregnant women with heart disease tends to occur in two distinct phases [3]. The first peak typically emerges towards the conclusion of the second trimester, coinciding with the point where cardiovascular demands reach their peak. The second peak occurs during the peri/early postpartum period. As pregnancy progresses into its later stages, diagnosing heart failure can be challenging, given that its characteristic signs and symptoms, such as sinus tachycardia, shortness of breath during physical activity, and peripheral edema, may closely resemble normal findings in late pregnancy.

Critical aortic stenosis (AS) is uncommon in women of childbearing age but may carry a significant risk of maternal mortality [5]. Left ventricular failure and sudden death are the major complications, and infective endocarditis is also a potential problem. In this type of valvular disease, the stroke volume is fixed, and patients lack the capacity to meet pregnancy-associated demands for increased cardiac output. Maintaining adequate systemic vascular resistance and blood pressure in these patients is crucial to ensure sufficient blood flow across the stenotic aortic valve. Any significant drop in blood pressure could theoretically lead to hemodynamic instability and potentially precipitate symptoms such as confusion, tachycardia, and other signs of cardiovascular compromise [6].

A significant discussion surrounds the ideal approach to anesthetic management for these patients. Some experts advocate for the cautious application of regional techniques, while others contend that general anesthesia is the preferred option [6].

The pathophysiological characteristics of aortic stenosis dictate the hemodynamic objectives, irrespective of the anesthesia method employed. Regional anesthesia can lead to a swift and unmitigated reduction in systemic vascular resistance (SVR), leading to a decline in blood pressure, coronary blood flow, and the onset of tachycardia [7]. In the case of typical patients, a decrease in SVR can be compensated for by increasing both stroke volume and heart rate. However, individuals with AS possess a fixed stroke volume, and their reliance on augmenting cardiac output rests mainly on elevating heart rate. Nevertheless, the presence of severe tachycardia is undesirable and carries potential risks.

In hindsight, if the patient had been proposed for an elective caeserean section and there had been a more careful and timely preoperative assessment, the case would probably have had a different outcome. Although the appearance of a heart murmur during pregnancy is physiological, this high-grade finding, together with the patient's complaints, would have been a sufficient indication for a cardiology assessment. Ideally, she would have gotten a transthoracic echocardiogram preoperatively and be referred to a tertiary center for the delivery with the knowledge of the cardiothoracic team.

Management of the parturient with high-risk cardiovascular disease ideally involves a high-risk pregnancy heart team that includes cardiologists, obstetricians, and anesthesiologists to develop an individualized management plan. Interdisciplinary communication and preparation are critical since peripartum complications may require urgent intervention.

Misoprostol is typically avoided in women with cardiovascular disease unless the benefits are likely to outweigh the risks, like in the case of uterine atony with hemorrhage. Although vaginal misoprostol has not been shown to affect maternal hemodynamics in healthy women, there are few reports of cardiovascular events after misoprostol administration. Misoprostol may have contributed to the symptoms, but the underlying cardiac condition appears to have been the main driver of the clinical presentation.

In this case report, it's important to emphasize that while these symptoms might have multiple potential causes, the differential diagnosis was critical for accurate diagnosis and appropriate management. In this case, the diagnosis of severe aortic stenosis played a central role in understanding the patient's clinical presentation and determining the treatment course.

Additionally, the potential advantage of bedside ultrasound is noteworthy. Using this non-invasive diagnostic approach could have expedited the confirmation of severe aortic stenosis and, ideally, provided a less invasive alternative to computed tomography, potentially averting the need for a more invasive examination for the patient.

The successful outcome in this challenging case highlights the potential for improved patient care and outcomes when healthcare professionals work in tandem to address complex clinical scenarios, emphasizing the necessity of interdisciplinary communication, medical expertise, and comprehensive clinical evaluation.

## Conclusions

Pregnancy is characterized by a period of profound physiological flux, placing significant demands on the cardiovascular system. These physiological adaptations can accentuate preexisting cardiac conditions or uncover latent cardiovascular concerns. Among these conditions, severe aortic stenosis assumes a distinctive role, requiring vigilant surveillance and collaborative care from a multidisciplinary team.

The anesthesia team plays a central and pivotal role in ensuring the safe healthcare provision to these patients. While heart disease has emerged as the primary contributor to maternal mortality, instances of critical aortic stenosis in pregnant individuals remain infrequent. Managing severe aortic stenosis poses a significant threat to maternal well-being and life, necessitating meticulous planning and a collaborative team strategy.
